# Designing Ternary Chiral DES to Enhance Enantioselectivity

**DOI:** 10.1021/acsomega.5c07420

**Published:** 2025-10-28

**Authors:** Hayden Teague, Ashton Lake, Todd A. Hopkins

**Affiliations:** Department of Chemistry and Biochemistry, 4065 Butler University, 4600 Sunset Avenue, Indianapolis, Indiana 46208, United States

## Abstract

Deep eutectic solvents (DES) are mixtures of two or three components that have a freezing point lower than that of the components. The properties of DES are tunable through the choice and ratio of the components. Three-component or ternary DES increase the flexibility to control the properties compared to binary DES. Two chiral ternary DES composed of tetraoctylammonium bromide (TOABr), tetrabutylammonium bromide (TBABr), (*R*)-ethyl mandelate ((*R*)-EM) or methyl-(2*R*)-2-hydroxy-3-phenylpropanoate ((*R*)-MHPP) were studied. Solid–liquid equilibrium measurements showed that only TOABr:TBABr:(*R*)-MHPP with 0.66 mole fraction of (*R*)-MHPP were liquid at room temperature, but TOABr:TBABr:(*R*)-EM mixtures were liquid over a wider range, 0.6–0.8, mole fraction of (*R*)-EM. The density, viscosity, conductivity, and polarity were measured for three TOABr:TBABr:(*R*)-MHPP and six TOABr:TBABr:(*R*)-EM mixtures and compared to the TBABr:(*R*)-MHPP and TBABr:(*R*)-EM. The enantioselectivity of the ternary DES was measured using circularly polarized luminescence (CPL) spectroscopic measurements of a luminescent lanthanide complex dissolved in the solvents. The ternary DES, TOABr:TBABr:(*R*)-MHPP and TOABr:TBABr:(*R*)-EM, exhibit higher enantioselectivity than the corresponding binary DES, TBABr:(*R*)-MHPP, TBABr:(*R*)-EM, and TOABr:(*R*)-EM.

## Graphical abstract

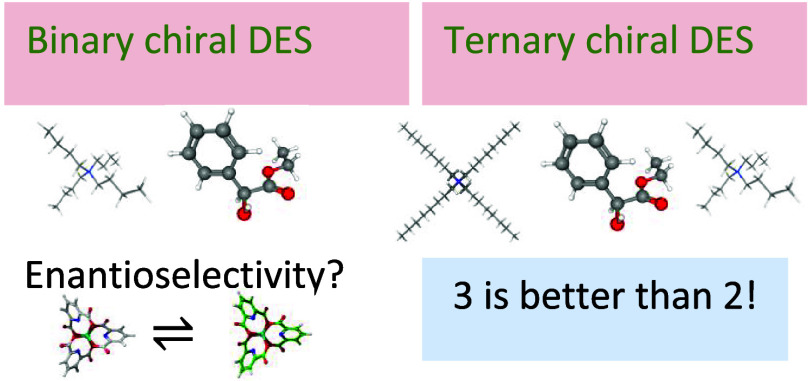

## Introduction

Deep eutectic solvents (DES) are mixtures of two or more components that have a freezing point lower than that of an ideal mixture of the components.^1–3^ This freezing point depression is the result of strong intermolecular interactions like hydrogen bonding, where the components are labeled as hydrogen bond acceptors (HBA) and hydrogen bond donors (HBD). Because they are typically prepared by simply mixing the components without a purification step, this atom economy makes them more likely to be green solvents.^4–7^ DES have physicochemical properties, such as low vapor pressure, conductivity, viscosity, and hydrophobicity, that are dictated both by the components and the molar ratio of those components.^1,2,8^ Therefore, the properties of DES are tunable through the choice of HBA, HBD, and ratio, and there have been several machine learning based methods developed to facilitate the discovery of DES.^9,10^ This tunability means that DES are used in a significant number of applications, including battery technology,^11–14^ pharmaceutical delivery,^15–17^ and waste processing.^18–22^

If one or more of the components are chiral, the resulting DES is a chiral solvent with applications in asymmetric synthesis,^23–27^ chiral separations,^28–31^ and chiral light-emitting materials.^32–35^ The tunability of the properties of chiral DES can be exploited to improve their chiral recognition and enantioselectivity of the solvents. The vast majority of reported DES are binary mixtures with the choice of HBA (i.e., ionic vs nonionic) and HBD as the variables to tune the properties.^1^ There are an increasing number of studies that use water or other molecular solvents as a third component to alter the structural and dynamic properties of the DES.^36–42^ However, there are fewer examples of exploiting the flexibility of three-component DES with some combination of HBAs and HBDs.^43–50^ Given the diversity of HBAs (e.g., tetraalkylammonium salts, metal chlorides, nonionic organic molecules) and HBDs, ternary mixtures open up an extremely large number of possible combinations to tailor DES properties.

This study involves ternary mixtures that contain two HBAs, tetrabutylammonium bromide (TBABr) and tetraoctylammonium bromide (TOABr), and a chiral HBD. The chiral HBDs studied are constitutional isomers methyl-(2*R*)-2-hydroxy-3-phenylpropanoate ((*R*)-MHPP) and (*R*)-ethyl mandelate ((*R*)-EM) with structures shown in Figure 1. The (*S*)- enantiomer of ethyl mandelate is commercially available, but the study will focus only on the (*R*) enantiomers. Previous screening of chiral DES shows that both HBDs form liquids with TBABr, 1:2 TBABr:(*R*)-MHPP, and 1:2 TBABr:(*R*)-EM.^51^ Since many DES exhibit structural and dynamic heterogeneity,^52–57^ the addition of a third hydrophobic component, such as TOABr, should increase the possibility of creating polar and nonpolar domains within the DES. The HBA, TBABr, and the HBDs (*R*)-EM and (*R*)-MHPP are not hydrophilic (logP 0.89, 1.5, 1.5, respectively^58–60^ ), but have very similar polarity, where the HBA, TOABr, is much more hydrophobic (log *P* = 4.5 ^61^) than any of the other components in Figure 1. Ternary mixtures of TOABr:TBABr: (*R*)-EM/MHPP form DES that allow the study of mixed polar and nonpolar HBAs on the enantioselectivity and physical properties of these chiral DES. Ternary DES may exhibit a “solvophobic” effect,^62,63^ where unfavorable interactions with the nonpolar HBA, TOABr, have the potential to increase the interaction between polar chiral solute and the polar chiral components of the DES, (*R*)-EM or (*R*)-MHPP, which could increase the enantioselectivity.

**Figure 1:**
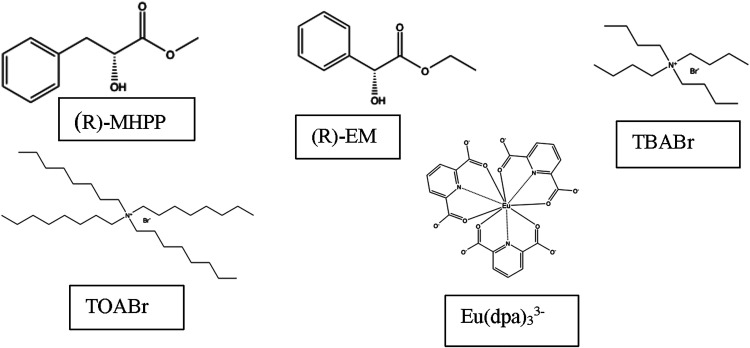
Structures of the HBAs, chiral HBDs, and the europium complex.

In this study, the enantioselectivity of the chiral DES is measured through circularly polarized luminescence (CPL) spectra induced by the solvent on a luminescent lanthanide complex, Eu­(dpa)_3_^3–^ (where dpa = 2,6-pyridine dicarboxylate anion), which is shown in Figure 1. CPL spectroscopy is the differential emission of left vs right circularly polarized light. Since Eu­(dpa)_3_^3–^ exists as a rapidly interconverting racemic mixture of Λ- vs Δ- enantiomers, without a chiral perturbation, the CPL signal is zero. When added to a chiral solvent that exhibits enantioselectivity, such as chiral DES, the Eu­(dpa)_3_^3–^ population becomes nonracemic, which results in a nonzero CPL signal.^35,64^ The sign and magnitude of the CPL signal are measures of the handedness and degree of enantioselectivity demonstrated by the chiral DES, respectively. The CPL spectra of Eu­(dpa)_3_^3–^ dissolved in the binary vs ternary DES show if the enantioselectivity is impacted by the presence of an additional nonpolar HBA, TOABr.

## Experimental Section

### DES Preparation

The chemicals used in this study, (*R*)- and (*S*)-ethyl mandelate, tetrabutylammonium bromide, tetraoctylammonium bromide, Methyl-(2*R*)-2-hydroxy-3-phenylpropanoate, Nile red, 4-nitroaniline, and *N*,*N*-diethyl-4-nitroaniline, were purchased from Combi-blocks or VWR and used without further purification. The tetraalkylammonium salts were stored in a vacuum desiccator before use in mixture preparation. Mixtures were prepared by adding the correct molar ratios of each of the components to a sample vial and stirring under heat at temperatures <50 °C until a homogeneous liquid was formed (typically less than 1 h). Samples prepared for freezing point determination that are solid after heating were mixed in a mortar and pestle to ensure homogeneity of mixing. Water content of the mixtures was measured by volumetric Karl Fischer titration (Metroohm 870 KF Titrino Plus).

### Physical Measurements

The freezing points of mixtures were measured with a differential scanning calorimeter (DSC) (TA Instruments DSC 25). DSC samples were prepared by adding 2–15 mg of the mixture to an aluminum pan and lid. The DSC was operated with a typical heating and cooling rate of between 2 and 5 °C/min under a constant flow of nitrogen gas. The water content of the larger mass samples was measured immediately before measuring the density, viscosity, and conductivity. Densities were obtained by determining the mass of DES in a 1.105 or 1.134 mL glass pycnometer. The viscosity was measured with a Brookfield DV2T viscometer, and conductivity was measured using a conductivity meter (Thermo Scientific Orion Star A212). Conductivities and viscosities were measured over a 283–323 K temperature range using a circulating water bath to control the temperature. Kamlett-Taft parameters were determined by dissolving small quantities of the solvatochromatic dyes Nile red, 4-nitroaniline, and *N*,*N*-diethyl-4-nitroaniline in each of the DES and measuring the UV–vis spectra (Cary 60).

### Spectroscopy Measurements and Sample Preparation

All of the starting materials for preparing Eu­(dpa)_3_^3–^, including EuCl_3_·6H_2_O, tetrabutylammonium hydroxide solution, sodium hydroxide, and 2,6-pyridinedicarboxylic acid, were purchased from Sigma-Aldrich and used without further purification. The complexes were prepared according to a previous procedure.^35^ The EuCl_3_·6H_2_O was dissolved in water, and three equivalents of sodium hydroxide was added to precipitate Eu­(OH)_3_, which was filtered and added to an aqueous solution of a slight excess of three equivalents of 2,6-pyridinedicarboxylic acid. Three equivalents of tetrabutylammonium hydroxide was added dropwise to precipitate (TBA)_3_Eu­(dpa)_3_, and the water was removed under vacuum. The precipitate was washed with water and filtered to leave an oily, white solid. CPL samples were prepared by dissolving small quantities of [TBA]_3_Eu­(dpa)_3_ in ∼1.5 g of the mixtures to give concentrations of (1–4) × 10^–6^ molal. The CPL and luminescence spectra of Eu­(dpa)_3_^3–^ samples dissolved in the DES were measured using a custom-built spectrometer described previously.^33,34^

## Results and Discussion

### TOABr:TBABr:(*R*)-MHPP

To find what molar ratios of the ternary mixture, TOABr:TBABr:MHPP, will be liquids at room temperature, the freezing points of multiple combinations were measured by DSC. Figure 2 shows a partial three-component solid–liquid phase diagram for TOABr:TBABr:MHPP. Mixtures with higher molar ratios (>40%) of TOABr were not measured because the freezing points are likely well above room temperature, 293 K, like the higher molar ratios of TBABr. Almost all of the ratios on the plot have freezing points above room temperature, except for the mixtures that are 66% MHPP. In fact, there does not seem to be a eutectic point in Figure 2, but more like a “eutectic valley” involving several ratios with 66% MHPP. Other than the mixtures in the eutectic valley, the remaining ternary mixtures are solid at room temperature.

**Figure 2:**
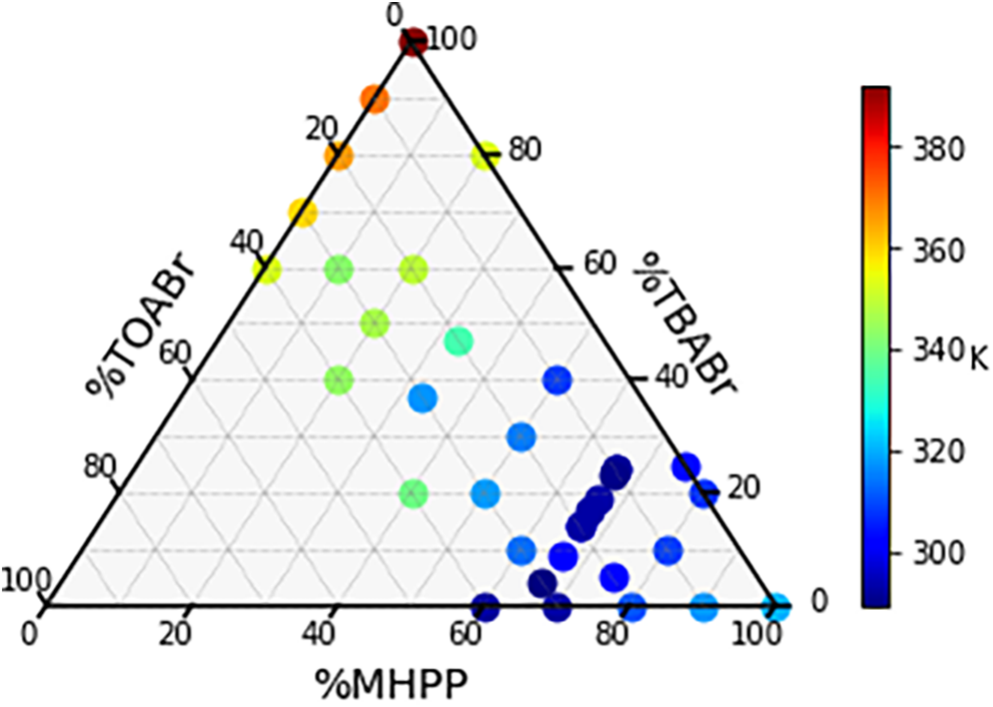
Solid–liquid equilibrium phase diagram for the three-component mixture of TOABr, TBABr, and MHPP. Freezing temperatures are represented by the color scale provided.

The lowest measured freezing point was 287 K for the 1:1:4 (0.17:0.17:0.66) TOABr:TBABr:MHPP, and there were two mixtures with freezing points <293 K, 1:2:6 and 1.5:1.9:6.6 TOABr:TBABr:MHPP. In principle, the thermodynamics of mixing and activity coefficients of ternary and binary mixtures can be determined using eq 1.

1 $$\ln\left(\gamma_{A}x_{A}\right)=\frac{{\Delta H}_{fus}}{R}\left(\frac{1}{T_{fus}}-\frac{1}{T}\right)$$

where γ_A_ and x_A_ are the activity coefficient and mole fraction of component A, *R* is the gas constant, *T*_fus_ and Δ*H*_fus_ are the freezing point and enthalpy of fusion of the component A, and *T* is the freezing point of the mixture. This equation is written without the contribution from the change in heat capacity upon melting (Δ_fus_ *C*_p_), because it is difficult to measure experimentally and typically makes a negligible contribution.^1^ The Δ*H*_fus_ and *T*_fus_ for TBABr are reported in the literature.^65^ Since the values of TOABr and MHPP have not been reported, the *T*_fus_ for TOABr and MHPP was determined by DSC, and Δ*H*_fus_ was determined by integrating the DSC peak (Figure S2). All of the thermodynamic values used in eq 1 are shown in the Supporting Information, Table S1. The activity coefficients of the four mixtures determined using eq 1 are shown in Table 1. The activity coefficients in all four mixtures are <1 for both TOABr and MHPP, indicating favorable mixing interactions (DES-like). The activity coefficients for TBABr in the four mixtures are ≥1, indicating unfavorable or ideal mixing. This suggests a preference for the interaction of the MHPP with the more hydrophobic HBA, TOABr. Using the method proposed by Panzer et al.,^66^ a dimensionless molar excess Gibbs free energy is determined for each mixture in Table 1 according to eq 2:

2 $$\frac{G^{E}}{\mathrm{RT}}=\sum x_{i}\;{\ln\gamma }_{i}$$

where *x_i_* is the mole fraction and γ*_i_* is the activity coefficient of the ith component, *G* ^E^ is the molar excess Gibbs free energy. Three of the four mixtures in Table 1 have *G* ^E^/RT < −0.33, which means they meet the criterion to be classified as DES. Only the 1:2.4:6.6 TOABr:TBABr:MHPP mixture does not meet the criterion, but it also has a freezing point that is slightly higher than room temperature at 294 K.

**Table 1:** Activity Coefficients for Ternary Mixtures with MHPP

TOABr:TBABr:MHPP	γ_TOABr_	γ_TBABr_	γ_MHPP_	G^E^/*RT*
1:1:4	0.14	1.16	0.64	–0.60
1:2:6	0.26	0.93	0.70	–0.40
1:2.4:6.6	0.35	0.97	0.78	–0.27
1.5:1.9:6.6	0.22	1.20	0.76	–0.37

Table 2 shows the water content, density, viscosity, conductivity, and Kamlet-Taft parameters for three ternary DES of TOABr:TOABr:MHPP at 293 K. The data for the binary 1:2 TBABr:MHPP is also shown for comparison. In all DES, the weight % of water is very low, which is consistent with the hydrophobicity of the HBAs. It is important to note that the mole fraction of MHPP (0.66) is equivalent in all of these DES. The densities and conductivities of the ternary DES are very similar to each other but decrease compared with those for the binary mixture. This is the result of adding bulkier HBA, TOABr, to the mixture. There is some variability in the measured viscosities, but viscosity is also more sensitive to the water content than other measurements. The viscosity and conductivity measurements over the 288-318 K temperature range are shown in the Supporting Information (Figure S3).

**Table 2:** Physical Properties for Mixtures with MHPP

					Kamlet-Taft		
TOABr:TBABr:MHPP	wt % water	density^a^ (g/mL)	viscosity^a^ (mPa·s)	conductivity^a^ (uS/cm)	α	β	π*
1:1:4	0.49	1.05	748	25	0.60	0.84	0.93
1:2:6	0.22	1.07	1041	36	0.52	0.85	0.96
1.5:1.9:6.6	0.10	1.05	943	26	0.59	0.87	0.95
0:1:2	0.02	1.15	1015	63	0.61	0.78	1.04

a Measured at 293 K experimental uncertainties: density = ±0.01 g/mL, viscosity = ±5 mPa·s, conductivity = ±1 uS/cm, α = ± 0.01, β = ±0.07, and π* = ±0.01.

Table 2 also shows the Kamlet-Taft parameters^67,68^ measured for the three ternary DES and the 1:2 TBABr:MHPP binary DES. All three parameters are almost identical for each of the ternary DES. There is not a large variation of molar ratios in this mixture that are liquid <293 K, which may be the reason that all of them have similar solvent polarity. Comparison with the binary mixture does show some differences. The hydrogen bond basicity, β, is larger in the ternary DES than the binary mixture even though the Br^–^ (the hydrogen bond acceptor) mole ratio is identical in all four DES, and the polarizability, π*, is smaller for ternary vs binary DES. The different HBAs are impacting the solvent properties. However, the hydrogen bond acidity, α, is identical for the ternary and binary DES, which may indicate that this is solely a function of the mole fraction of HBD, MHPP.

Figure 3 shows the CPL and average luminescence spectra for the ^5^D_0_ → ^7^F_0–2_ transitions of Eu­(dpa)_3_^3–^ dissolved in 1.5:1.9:6.6 TOABr:TBABr:(*R*)-MHPP. The (*S*) enantiomer of MHPP is not commercially available, so this study focused only on the (*R*) enantiomer. The average luminescence shows the typical spectral pattern for Eu­(dpa)_3_^3–^ with a large peak at 615 nm for the ^5^D_0_ → ^7^F_2_ transition, two smaller peaks at 592 and 594 nm for the ^5^D_0_ → ^7^F_1_ transition. The ^5^D_0_ → ^7^F_0_ (symmetry forbidden) transition at 580 nm is extremely weak. This indicates that the Eu­(dpa)_3_^3–^ structure is stable when it is dissolved in the mixture. Additionally, the CPL spectra show that the ^5^D_0_ → ^7^F_2_ (positive) vs ^5^D_0_ → ^7^F_1_ (negative) transitions are of opposite sign, which is also characteristic of enantiomerically resolved Eu­(dpa)_3_^3–^ complexes.^33–35^ The spectra for all of the other DES showed identical spectral features and a CPL sign.

**Figure 3:**
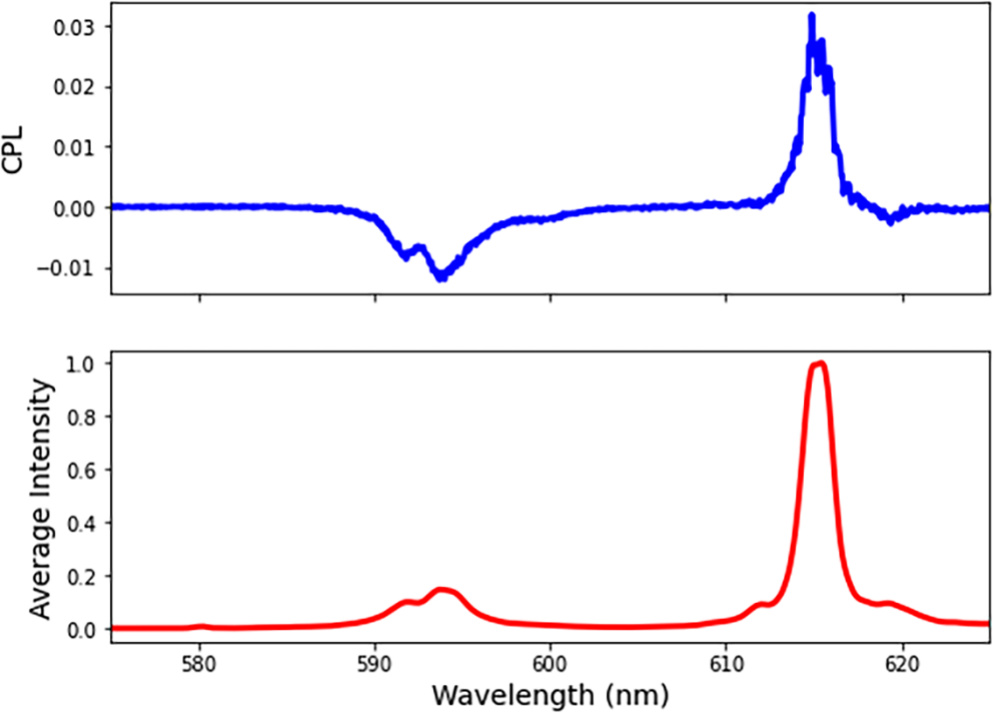
CPL (top blue) and average luminescence spectrum (bottom red) of the ^5^D_0_ → ^7^F_0–2_ transitions of Eu­(dpa)_3_^3–^ dissolved in 1.5:1.9:6.6 TOABr:TBABr:(*R*)-MHPP.

Table 3 shows the emission dissymmetry factors, *g*_em_(λ), determined for each of the DES. The emission dissymmetry factor is a measure of the degree of polarization of the emitted light given in eq 3

3 $$g_{\mathrm{em}}\left(\lambda \right)=\frac{2\left(I_{L}-I_{R}\right)}{\left(I_{L}+I_{R}\right)}=\frac{\mathrm{CPL}}{\mathrm{Ave}L}$$

where *I*_L_ and *I*_R_ are the intensities of left and right circularly polarized light, and Ave L is the average luminescence. In these experiments, the *g*_em_(λ) provides a measure of the shift in the racemization equilibrium of Λ- vs Δ-Eu­(dpa)_3_^3–^ by the chiral DES, where the sign of *g*_em_(λ) indicates the sense and the magnitude quantifies the enantioselectivity.^35,64^ All of the DES, ternary and binary, in Table 3 have *g*_em_(594 nm) < 0, which is the preference dictated by the handedness of the HBD, (*R*)-MHPP. Since the DES in Table 3 have the same mole fraction of (*R*)-MHPP, there is little variation of the dissymmetry factors for the ternary DES. The *g*_em_ for the ternary DES is slightly larger in magnitude (more negative) than the binary 1:2 TBABr:(*R*)-MHPP. Samples of binary TOABr:(*R*)-MHPP with Eu­(dpa)_3_ were solid when added to a cuvette so CPL and *g*_em_ could not be measured. This shows that the addition of TOABr does not change the preference but does increase the enantioselectivity compared with the binary DES.

**Table 3:** Emission Dissymmetry Factors for Eu­(dpa)_3_^3–^ in TOABr:TBABr:(*R*)-MHPP DES

TOABr:TBABr:(*R*)-MHPP	*g*_em_(594 nm)^a^
1:1:4	–0.067
1.5:1.9:6.6	–0.070
1:2:6	–0.070
0:1:2	–0.062

a Uncertainty ±0.002.

### TOABr:TBABr:(*R*)-EM

The other ternary mixture studied has (*R*)-EM as the HBD in place of (*R*)-MHPP. MHPP and EM are constitutional isomers but have very different properties as HBDs in mixtures. Figure 4 shows a partial ternary solid–liquid phase diagram for TOABr:TBABr:(*R*)-EM. As is the case in Figure 2, high molar ratios of TOABr were not measured because the freezing points of these mixtures are well above room temperature (293 K). Unlike the MHPP mixtures, all of the mixtures with 60–80% (*R*)-EM are liquids <293 K, and very few of the ternary (or binary) mixtures with 60–70% (*R*)-EM showed any freezing point outside of glass transitions ∼210 K. This makes it nearly impossible to determine a definitive “eutectic valley” or eutectic point for this mixture.

**Figure 4:**
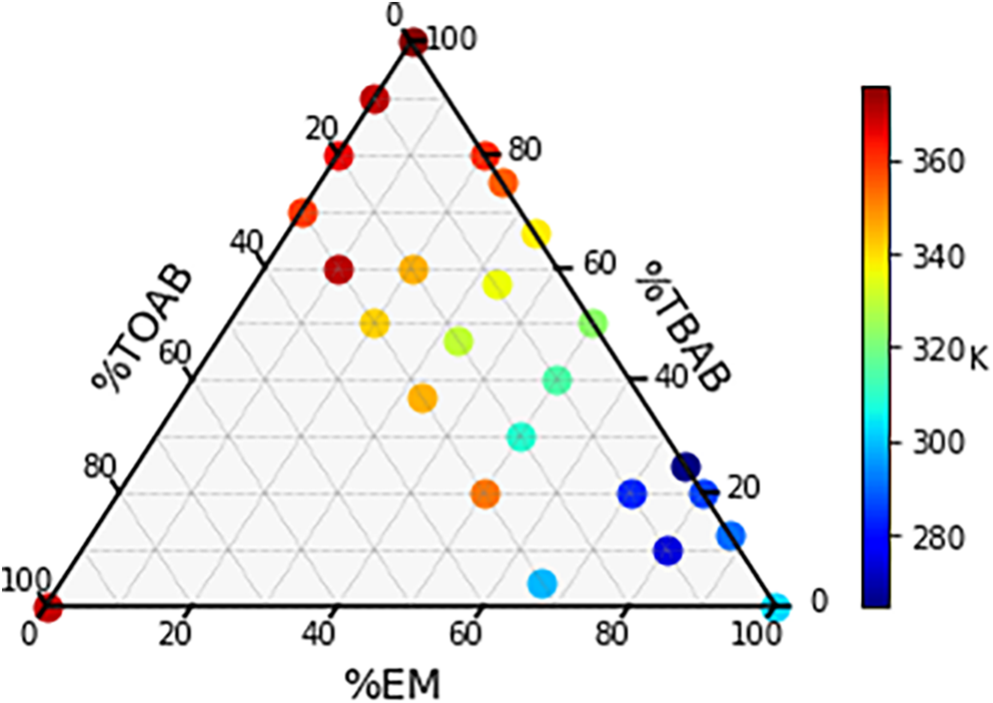
Solid–liquid equilibrium phase diagram for the three-component mixture of TOABr, TBABr, and (*R*)-EM. Freezing temperatures are represented by the color scale provided (note that the color scheme differs from Figure 2).

Only two of the ternary mixtures had measurable freezing points to use to determine activity coefficients (eq 1) and dimensionless molar excess Gibbs free energy (eq 2). The 1:1:8 TOABr:TBABr:(*R*)-EM mixture has a freezing point of 274 K, which gave γ_TOABr_ = 0.11, γ_TBABr_ = 1.44, γ_EM_ = 0.53, and *G* ^E^/RT = −0.69. The 1:2:7 TOABr:TBABr:(*R*)-EM mixture has a freezing point of 282 K with γ_TOABr_ = 0.18, γ_TBABr_ = 0.88, γ_EM_ = 0.79, and *G* ^E^/RT = −0.36. Both mixtures meet the threshold to be considered DES. By many established definitions, only one mixture of components is the DES,^1^ but for simplicity of language, all of the room temperature liquid ternary mixtures of TOABr:TBABr:(*R*)-EM will be labeled DES.

There are considerably more low-temperature liquid ternary DES with (*R*)-EM than (*R*)-MHPP as the HBD. Table 4 shows water content, density, viscosity, conductivity, and Kamlet–Taft parameters for six ternary DES 1:2:6, 1:1:4–8 TOABr:TBABr:(*R*)-EM and the binary DES 1:2 TBABr:(*R*)-EM. Each of the DES have relatively low weight percent water. The density of the ternary DES is the same within experimental uncertainty but is also less than the density of the 1:2 TBABr:(*R*)-EM. Similar to the MHPP DES (Table 3), the addition of the larger HBA, TOABr, decreases the density of the DES. As the ratio of (*R*)-EM increases, the viscosity decreases and the conductivity increases. Complete temperature-dependent viscosity and conductivity over the 288–318 K are shown in the Supporting Information (Figure S5). The hydrogen bond acidity, α, increases and the hydrogen bond basicity, β, decreases as the molar ratio of HBD, (*R*)-EM, increases in the ternary DES. The polarizability, π*, is independent of the molar ratios in the ternary DES. Both β and π* are smaller in the ternary DES than in the binary 1:2 TBABr:(*R*)-EM. For the equivalent mole fraction of (*R*)-EM (1:2:6, 1:1:4 vs 1:2), α is larger for the binary vs ternary DES, which contrasts with the DES with MHPP as the HBD (Table 2).

**Table 4:** Physical Properties for Mixtures with (*R*)-EM

					Kamlet–Taft		
TOABr:TBABr:(*R*)-EM	wt % water	density^a^ (g/mL)	viscosity^a^ (mPa·s)	conductivity^a^ (uS/cm)	α	β	π*
1:2:6	0.32	1.06	704	47	0.62	0.96	0.89
1:1:4	0.35	1.05	600	31	0.64	0.96	0.87
1:1:5	0.11	1.05	345	41	0.63	0.82	0.91
1:1:6	0.11	1.07	236	52	0.69	0.84	0.91
1:1:7	0.62	1.06	167	65	0.72	0.82	0.87
1:1:8	0.05	1.07	167	62	0.73	0.75	0.89
0:1:2	0.34	1.13	467	54	0.74	1.23	1.29

a Measured at 293 K experimental uncertainties: density = ±0.01 g/mL, viscosity = ±5 mPa·s, conductivity = ±1 uS/cm, α = ±0.01, β = ±0.07, and π* = ±0.01.

The CPL and luminescence spectra of Eu­(dpa)_3_^3–^ dissolved in the ternary DES of 1:2:6, 1:1:4–8 TOABr:TBABr:(*R*)-EM have the same spectral features as shown in Figure 3 for the MHPP ternary DES. An example of the CPL and luminescence spectra of Eu­(dpa)_3_^3–^ dissolved in 1:1:6 TOABr:TBABr:(*R*)-EM DES is shown in the Supporting Information (Figure S6). The sign of the CPL is also the same for (*R*)-EM as for (*R*)-MHPP, where the ^5^D_0_ → ^7^F_1_ transition is negative and the ^5^D_0_ → ^7^F_2_ transition is positive. Table 5 shows the emission dissymmetry factors (eq 3) determined for the six ternary TOABr:TBABr:(*R*)-EM DES and four binary DES, 1:2 and 1:4 TBABr:(*R*)-EM, 1:3 and 1:4 TOABr:(*R*)-EM. It is notable that *g*_em_ gets more negative as the ratio of (*R*)-EM increases.

**Table 5:** Emission Dissymmetry Factors for Eu­(dpa)_3_^3–^ in TOABr:TBABr:(*R*)-EM DES

TOABr:TBABr:(*R*)-EM	*g*_em_ (594 nm)^a^	TOABr:TBABr:(*R*)-EM	*g*_em_ (594 nm)^a^
0:1:2	–0.060	1:2:6	–0.072
0:1:4	–0.091	1:1:4	–0.100
1:0:3	–0.061	1:1:5	–0.099
1:0:4	–0.071	1:1:6	–0.117
		1:1:7	–0.120
		1:1:8	–0.122

a Uncertainty ± 0.002.

To more clearly and quantitatively compare *g*_em_ across ternary and binary DES, the molar ratios are converted to mole fractions and the *g*_em_ vs mole fraction of (*R*)-EM are shown in Figure 5. As shown in Table 5, the *g*_em_ gets more negative as *x*_EM_ increases. This shows that as the chiral HBD, (*R*)-EM increases so does the magnitude of the enantioselectivity, which is consistent with a previous study on sugar-based DES.^69^ Figure 5 also shows that the *g*_em_ is more negative (and enantioselective) for the ternary vs binary DES. This is demonstrated at three different mole fractions, *x*_EM_ = 0.66, 0.75, and 0.8. The *g*_em_ measured at *x*_EM_ = 0.66 also shows a decrease with an increase in the mole fraction of TOABr in the ternary DES. At *x*_EM_ = 0.80, the *g*_em_ is more negative for 0.2:0.8 TBABr:(R)-EM versus 0.2:0.8 TOABr:(*R*)-EM, which seems to indicate that the more polar binary DES shows better enantioselectivity. At *x*_EM_ = 0.75, the *g*_em_ is twice as negative for ternary vs the TOABr:(*R*)-EM. Collectively, the data in Figure 5 indicate that there is a synergistic effect to combining TOABr with TBABr that increases the enantioselectivity of the ternary vs binary DES. This is likely a confirmation of the “solvophobic” effect that mixing a nonpolar achiral HBA increases the enantioselectivity of a polar HBD of a polar chiral solute, like Eu­(dpa)_3_^3–^.

**Figure 5:**
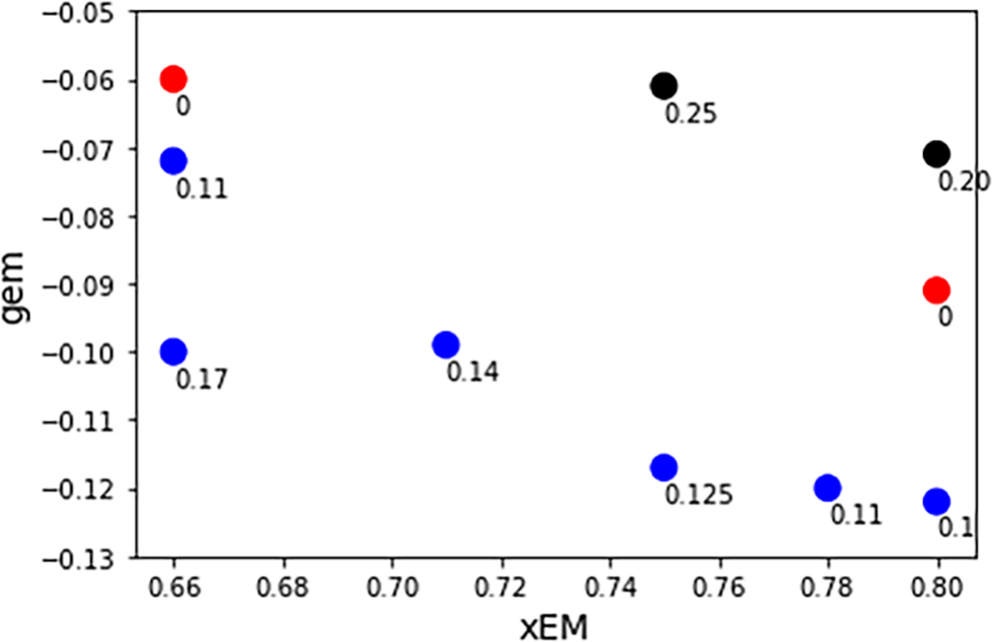
Dissymmetry factor vs mole fraction of (*R*)-EM for ternary TOABr:TBABr:(*R*)-EM (blue circles), TBABr:(*R*)-EM (red circles), and TOABr:(*R*)-EM (black circles). The data labels show the mole fraction of TOABr for each data point.

The *g*_em_ is a measure of how well the DES shifts the equilibrium to a nonracemic population of Λ- vs Δ-Eu­(dpa)_3_^3–^. The measured *g*_em_ can be related to an excited-state enantiomeric excess

4 $$\eta^{*}=\frac{[\Lambda^{*}]-\left[\Delta^{*}\right]}{[\Lambda^{*}]+\left[\Delta^{*}\right]}=\frac{g_{\mathrm{em}}\left(\lambda \right)}{g_{\mathrm{em}}^{\Lambda }\left(\lambda \right)}$$

where [Λ*] an [Δ*] are the excited-state populations of the enantiomers of Eu­(dpa)_3_^3–^, and *g*_em_^Λ^(λ) = 0.29 is the dissymmetry factor for an enantiomerically resolved population of Λ-Eu­(dpa)_3_^3–^.^64^ Assuming there is no enantioselective quenching, the ground state population of Λ and Δ are equal to the excited-state populations, and the excited-state and ground state enantiomeric excess are also equivalent. Therefore, the *g*_em_ and η* are direct measures of the enantioselectivity from DES solvation. Using eq 4, the 1:2 TBABr:(*R*)-EM gives η* = −0.21 which represents a population that is 60% Δ-Eu­(dpa)_3_^3–^ while 1:1:4 TOABr:TBABr:(*R*)-EM gives η* = −0.34, which increases to 66% Δ-Eu­(dpa)_3_^3–^.

## Conclusion

In this study, ternary chiral DES were prepared by mixing two HBAs and one HBD. The HBDs are two similar chiral molecules, (*R*)-MHPP and (*R*)-EM, and the HBAs include TBABr and the hydrophobic TOABr. Ternary solid–liquid equilibrium phase diagrams were measured for the mixtures with each HBD. Only mixtures that have a mole fraction of MHPP = 0.66 were liquid at room temperature. Where mixtures with (*R*)-EM mole fractions between 0.6 and 0.8 were liquid at room temperature. The ternary mixtures that are liquid at room temperature have molar excess Gibbs free energies that qualify them as DES. Because of the similarity in molar ratios, the three ternary DES, 1:1:4, 1:2:6, 1.5:1.9:6.6 TOABr:TBABr:(*R*)-MHPP, had very similar properties, including density, viscosity, conductivity, polarity, and enantioselectivity. There were six ternary DES, 1:2:6, 1:1:4–8 TOABr:TBABr:(*R*)-EM prepared. The differences in properties were primarily a function of the change in the ratio of the HBD, (*R*)-EM. Ternary DES with higher (*R*)-EM had lower viscosities, higher hydrogen bond acidity, and lower hydrogen bond basicity, and they exhibited higher enantioselectivities as measured by induced CPL. Ternary DES with TOABr:TBABr:(*R*)-EM had higher enantioselectivity than binary DES with TBABr:(*R*)-EM or TOABr:(*R*)-EM. The two HBAs with different polarities seem to have a synergistic effect on the enantioselectivity of the DES, and it justifies the strategy of using ternary mixtures to tune the properties of DES. This chiral “solvophobic” effect from ternary chiral DES could be applied to enhance asymmetric synthesis or chiral separations.

## Supplementary Material



## Notes

Includes DSC plots of pure components and mixtures of TOABr:TBABr: (*R*)-MHPP and TOABr:TBABr: (*R*)-EM, thermodynamic properties of all of the pure components, temperature-dependent viscosities, and conductivities for mixtures of TOABr:TBABr: (*R*)-MHPP and TOABr:TBABr: (*R*)-EM, CPL spectrum for Eu­(dpa)_3_^3–^ in 1:1:6 TOABr:TBABr:(*R*)-EM (PDF)
