# Supplementary material for: Designing Ternary Chiral DES to Enhance Enantioselectivity
Source: ACS Omega. 2025 Oct 28;10(44):52968–75. doi: 10.1021/acsomega.5c07420 (PMC12612977; doi:10.1021/acsomega.5c07420)
Supplement: Supplementary file 1 [file ao5c07420_si_001.pdf]

Supporting Information for

Designing ternary chiral DES to enhance enantioselectivity

Hayden Teague, Ashton Lake, and Todd A. Hopkins

Department of Chemistry and Biochemistry, Butler University, 4600 Sunset Avenue,

Indianapolis, IN 46208.

Table of Contents

1. DSC plots for TOABr:TBABr: (R)-MHPP mixtures
2. DSC plots for (R)-EM, (R)-MHPP, and TOABr
3. Thermodynamic properties of pure components
4. Temperature dependent viscosities and conductivities of TOABr:TBABr: (R)-MHPP mixtures.
5. DSC plots for TOABr:TBABr: (R)-EM mixtures
6. Temperature dependent viscosities and conductivities of TOABr:TBABr: (R)-EM mixtures.
7. CPL and luminescence spectra for $Eu(dpa)_3^{3-}$ dissolved in 1:1:6 TOABr:TBABr: (R)-EM

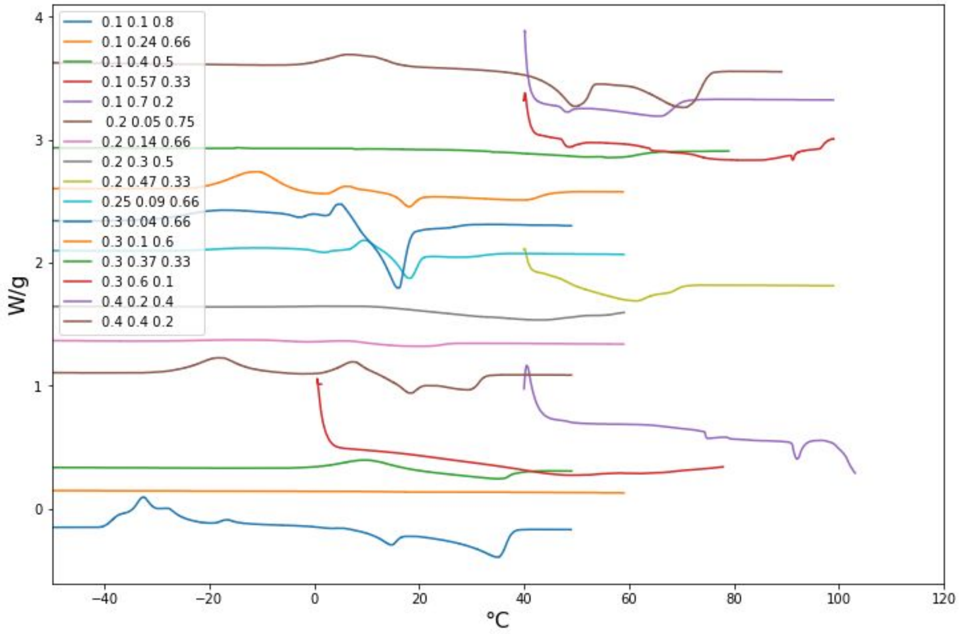

Figure S1. DSC plots for the heating curves for the TOABr:TBABr: (R)-MHPP mixtures.

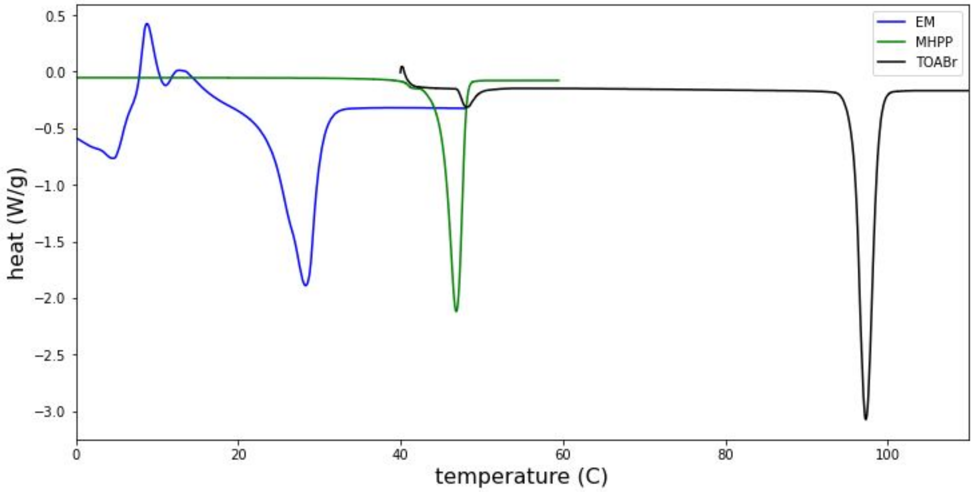

Figure S2. DSC plots of (R)-EM, (R)-MHPP, and TOABr.

Table S1. Thermodynamic properties of the pure components.

	Melting point (K)[a]	Enthalpy of fusion (kJ/mol)[a]
TBABr	377	16.15
TOABr	370	39.8
EM	302	20.88
MHPP	321	19.3

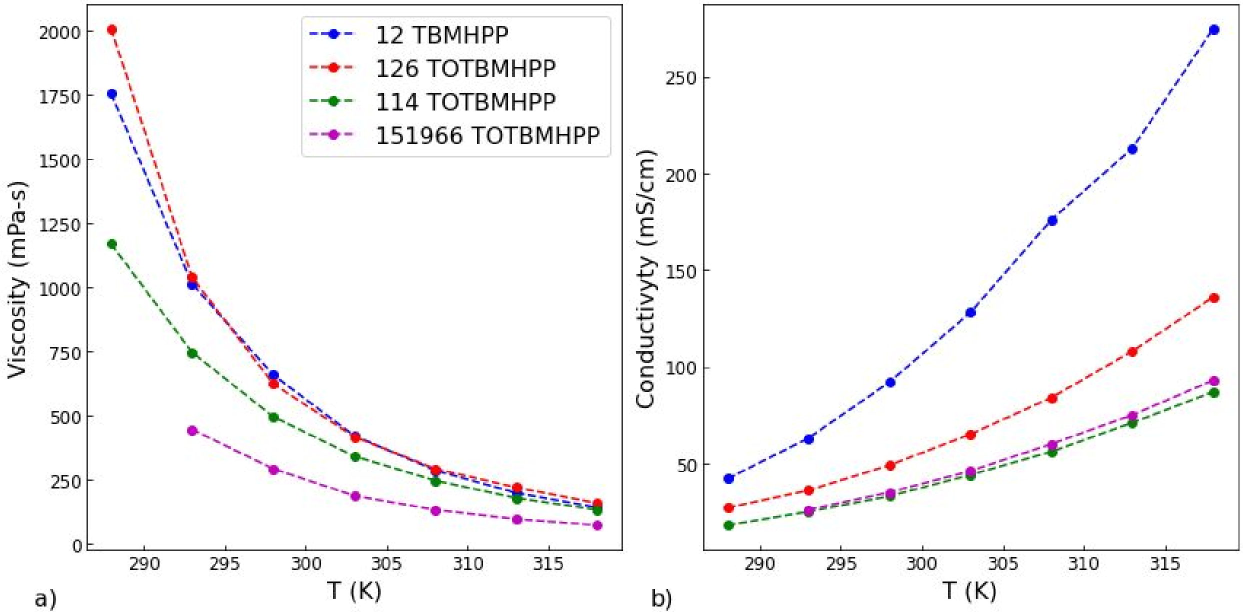

Figure S3. Temperature dependent viscosities and conductivities for TOABr:TBABr:MHPP mixtures.

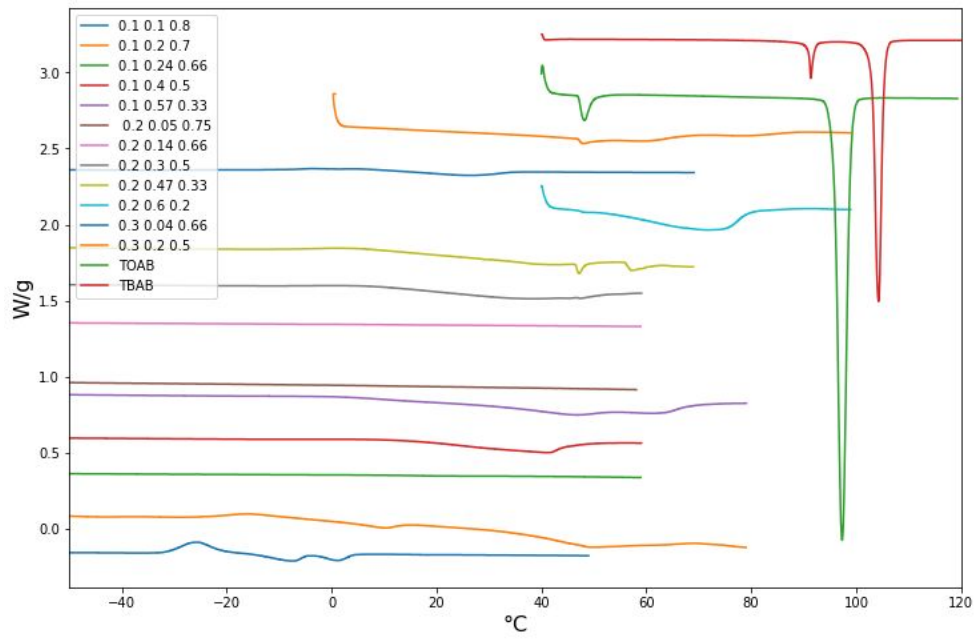

Figure S4. DSC plots for the heating cycle for TOABr:TBABr:R-EM. Most of the heating curves started at -70 ºC, but several start at higher temperatures.

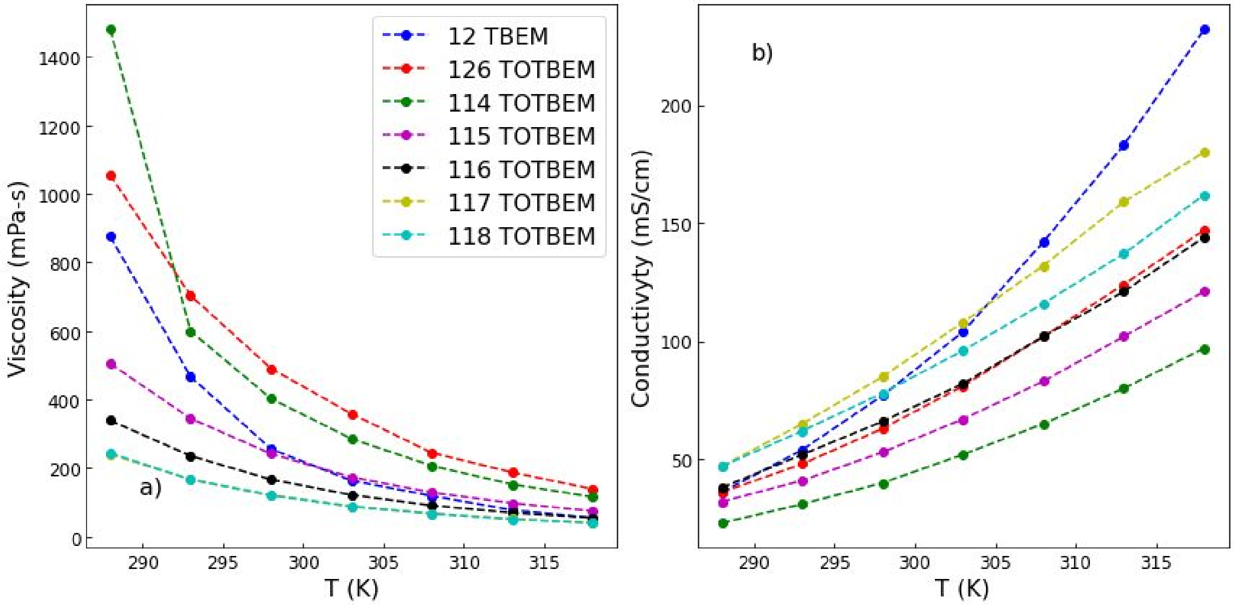

Figure S5. Temperature dependent a) viscosities and b) conductivities for TOABr:TBABr:(R)-EM mixtures.

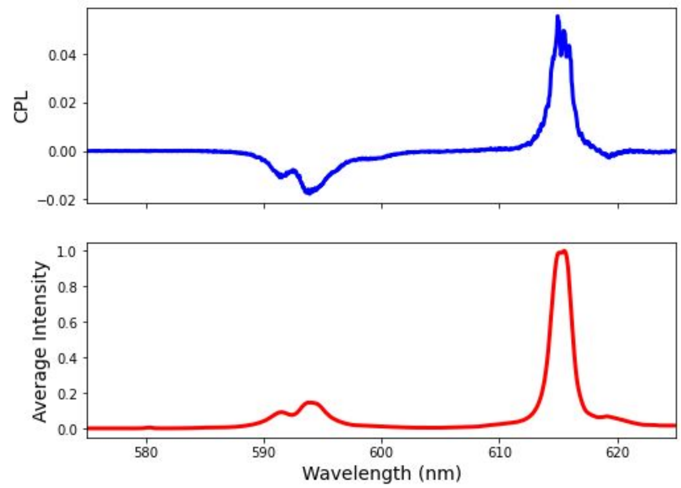

Figure S6. CPL (top blue) and average luminescence spectrum (bottom red) of the $^5D_0 \rightarrow {}^7F_{0—2}$ transitions of $Eu(dpa)_3^{3-}$ dissolved in 1:1:6 TOABr:TBABr:(R)-EM.
